# Supplementary material for: A Benzodiazepine-Derived Molecule That Interferes with the Bio-Mechanical Properties of Glioblastoma-Astrocytoma Cells Altering Their Proliferation and Migration
Source: Int J Mol Sci. 2025 Mar 19;26(6):2767. doi: 10.3390/ijms26062767 (PMC11943291; doi:10.3390/ijms26062767)
Supplement: Supplementary file 1 [file ijms-26-02767-s001.zip › ijms-3464710-supplementary.pdf]

Supplementary Materials for:

A benzodiazepine-derived molecule interferes with the bio-mechanical properties of glioblastoma-astrocytoma cells altering their proliferation and migration

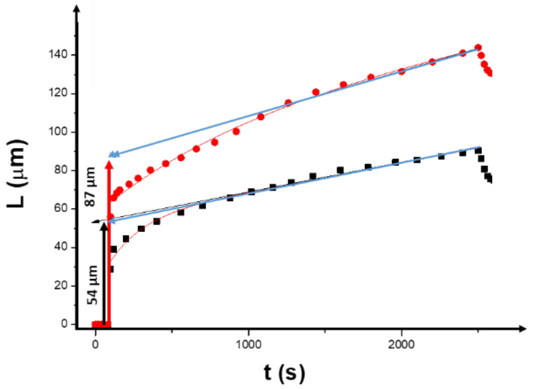

Figure S1: Examples of the evaluation of the intercept for t = 0 corresponding to $\delta = \frac{F}{k_1}$. One case for the control experiment (black squares) and one case for 1g 20 µM have been reported.

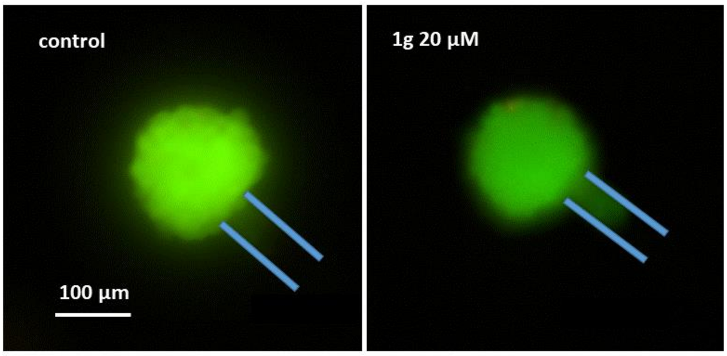

Figure S2: Live-dead staining (green and red, respectively) of spheroids undergoing an experiment of Micropipette Aspiration. In the case of both control and 1g treated cells, the large majority of cells are alive. The micropipette has been drawn for clarity.

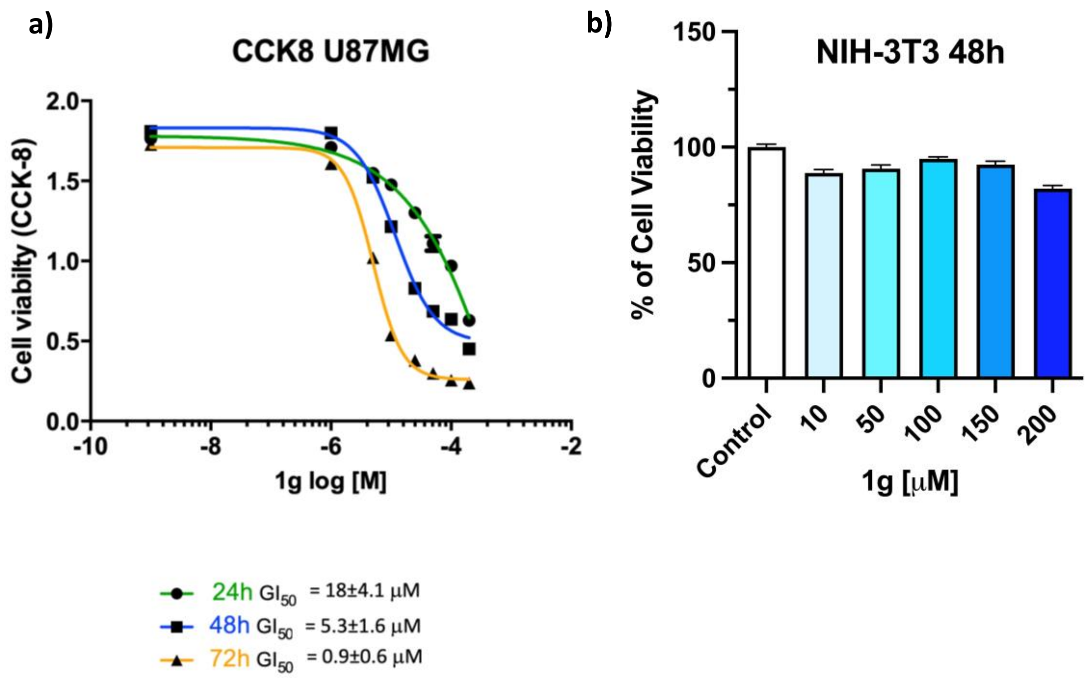

Figure S3: Cytotoxic effects of 1g on U87MG and NIH-3T3 cell lines. (a) U87MG were exposed to the indicated concentrations of 1g for 24, 48, and 72 hours. IC 50 was obtained from 3 independent experiments with 4 replicates each. (b) NIH-3T3 were exposed to 1g at the indicated concentration for 48 h. The values, expressed as the mean ± SD of three independent experiments (4 replicates each), are scaled to those in untreated cultures exposed continuously to 0.1% DMSO only and normalized to 100%

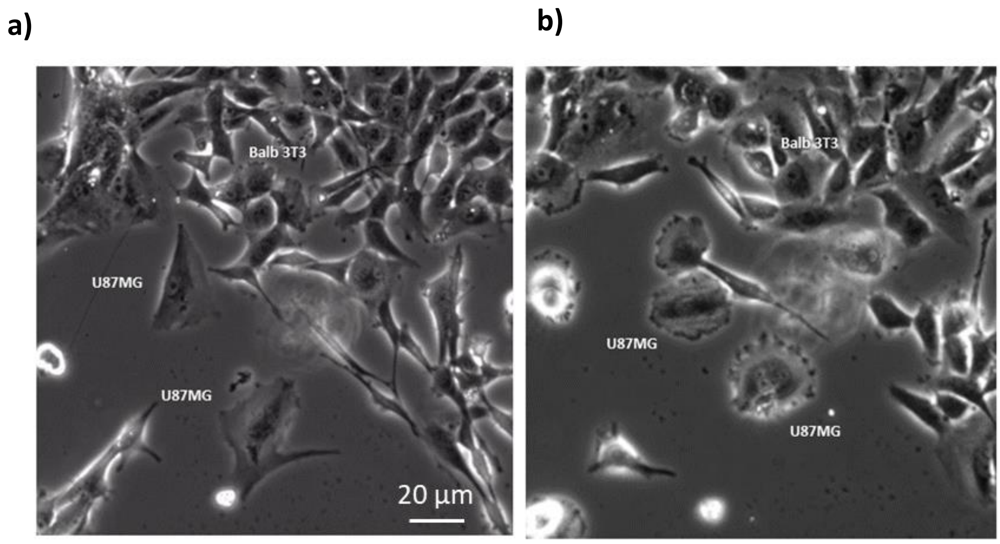

Figure S4: U87MG and NIH/3T3 co-culture. Before adding 1g to the culture medium both types of cells are elongated (a), after the insertion of 20 μM 1g (b) only U87MG cells get round whereas 3t3 cells have a negligible change of their shape.

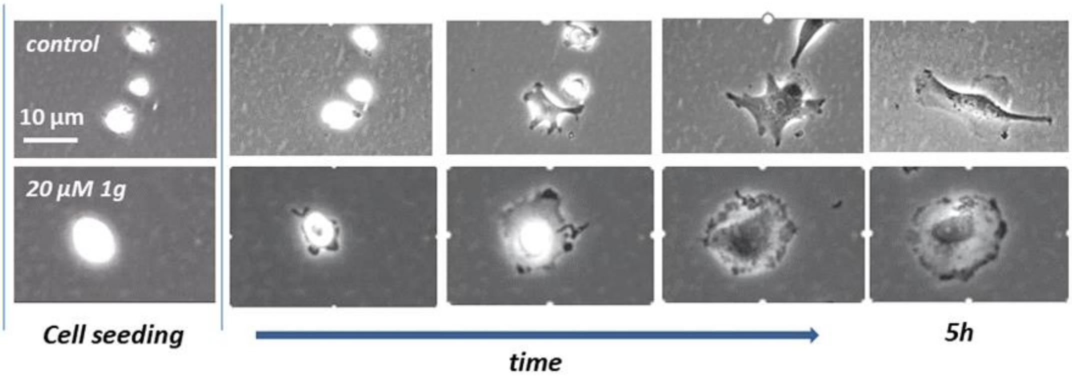

Figure S5: Seeding of cells in the absence and in the presence of 1g. In the absence of 1g, within 5 hours, cells spread on the surface but, if in the control case cells acquire a polarization and elongate, in the presence of 1g cells get round and they are not able to polarize.

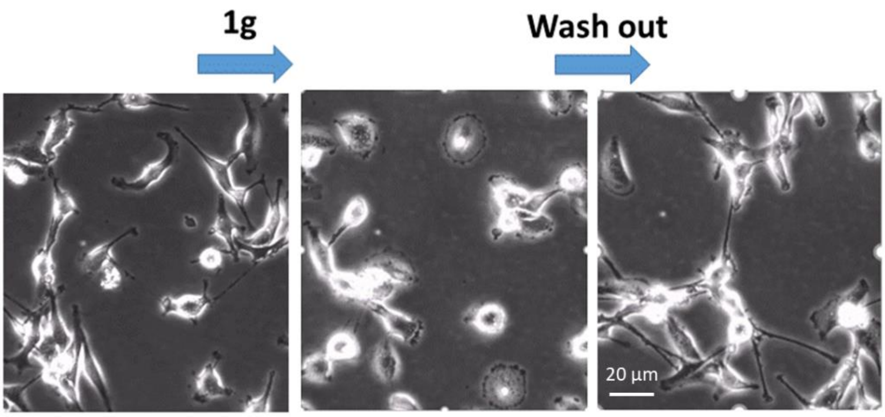

Figure S6: Wash-out experiment to show the reversibility of cell shape changes. The first image shows U87MG cells in their normal culture medium just before injecting 20 µM 1g in the medium; the second image refers to 1 hour after the injection of 1g just before changing the medium to remove 1g; the last image refers to the cells 2 hours after the removal of 1g in which it is evident that they acquire again a polarization.

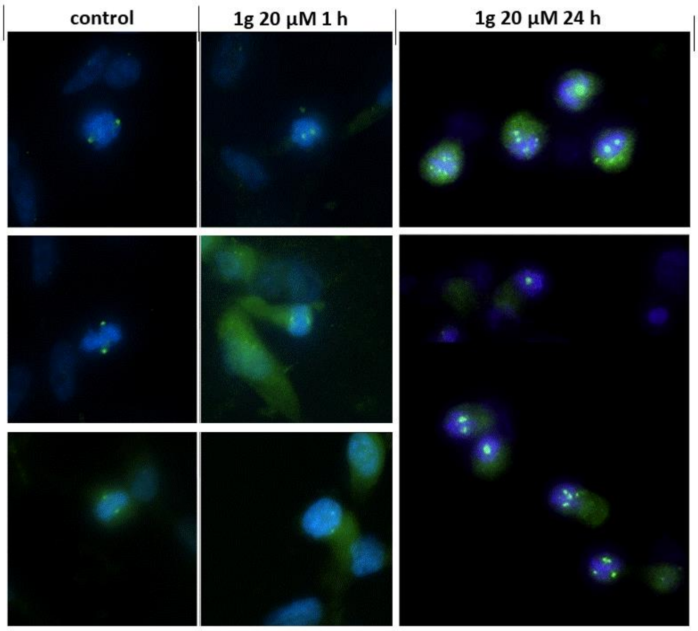

Figure S7: γ-tubulin immunofluorescence to assess the activity of Kif11 upon 1g injection. In control cells, the centrosomes, as marked by γ-tubulin, reach a symmetrical position along the diameter of the nuclear material. 1 h after the injection of 20 µM 1g, we start observing situations in which centrosomes (as seen by -tubulin staining) are not correctly positioned when the nuclear envelope appears as already destroyed. After 24 h more than two asters appear and the separation among the different asters is small compared to the normal distance of the two asters in control cells. The proper function of astral microtubules is strictly required for the correct positioning and separation of the centrosomes. In fact, interactions of microtubules with the cortical region are important to provide the force required for a proper positioning of centrosomes.

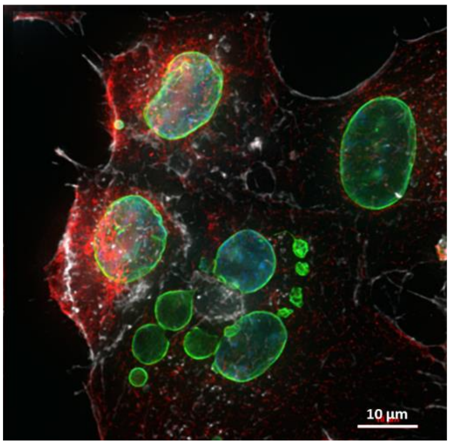

Figure S8: Formation of multinuclear structures. When cells flatten on the surface after an attempt to duplicate, many structures confined by lamin A-C appear. In the image microtubules are in red, actin in white and lamin A-C in green

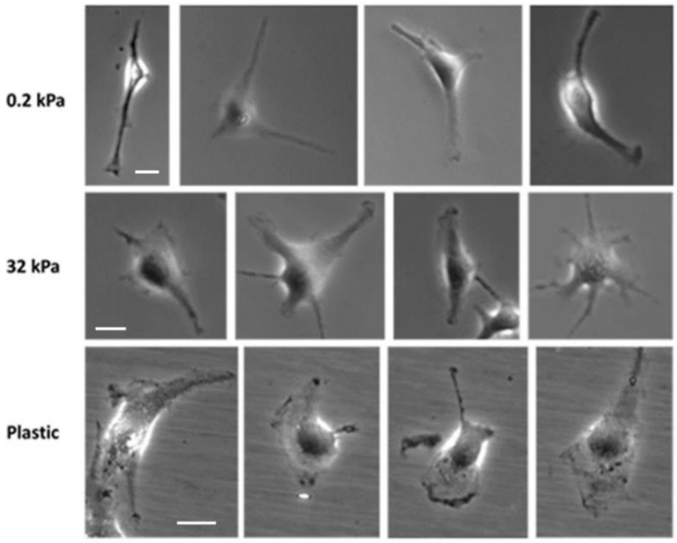

Figure S9: adhesion area of U87MG cells on substrates of different stiffness. The bar corresponds to 10 µm and it is the same for images on the same line

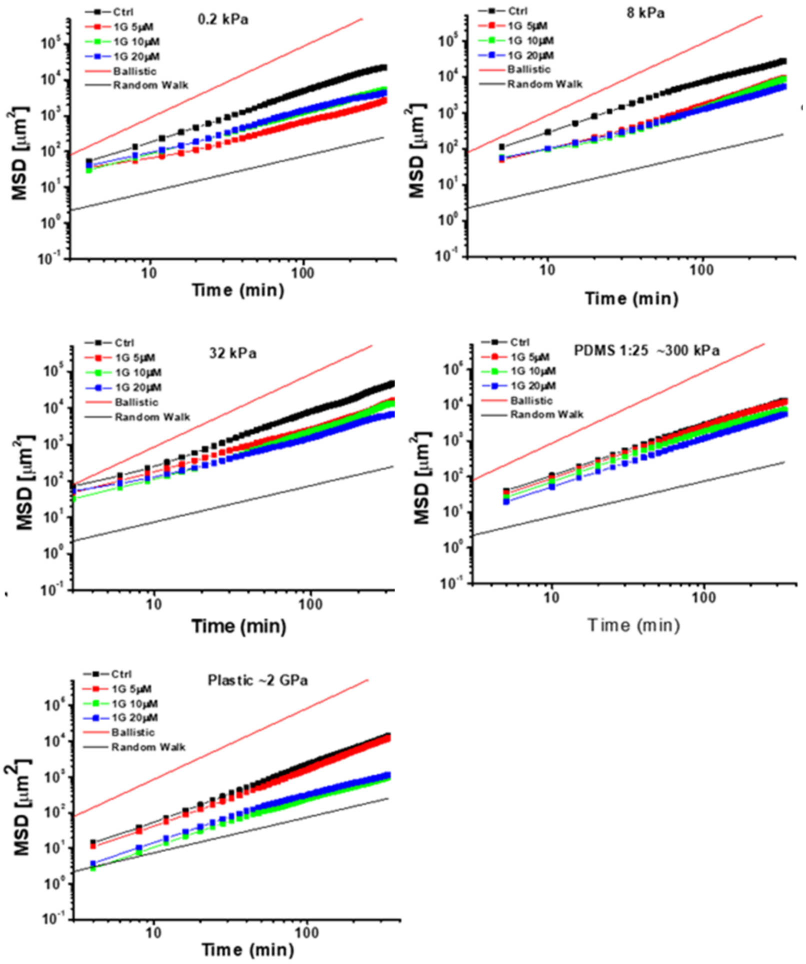

Figure S10: Plots of the MSD as a function of time for different 1g concentrations grouped according to the rigidity of the substrate. In each plot the trend of both the purely random walk behavior and of the ballistic behavior is reported.

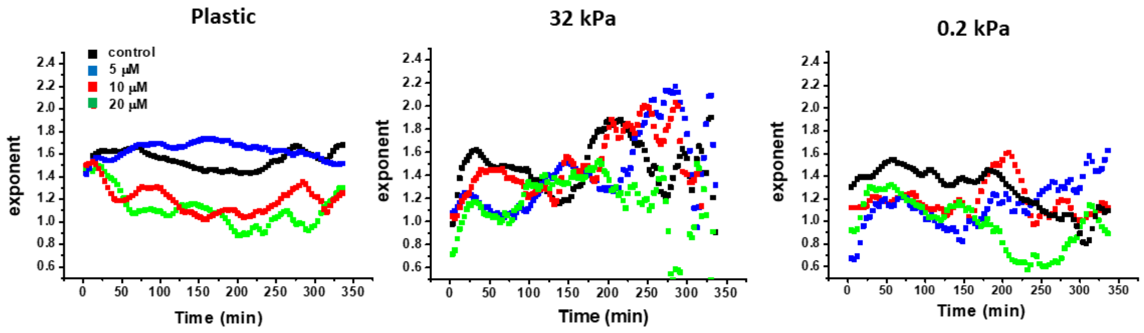

Figure S11: Exponent of the MSD function with respect to time for three different values of the substrate rigidity. The exponent is obtained considering the relationship MSD $\propto t^{\alpha}$ Log(MSD) $\propto$ αLog(t)

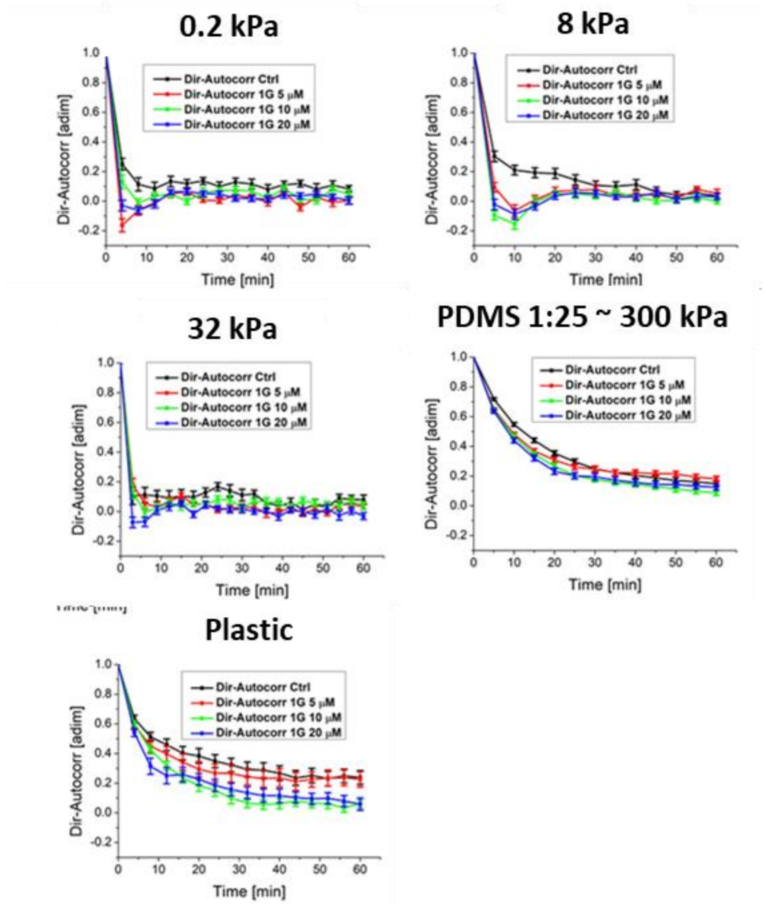

Figure S12: Direction autocorrelation function on substrates of different rigidity as a function of 1g concentration

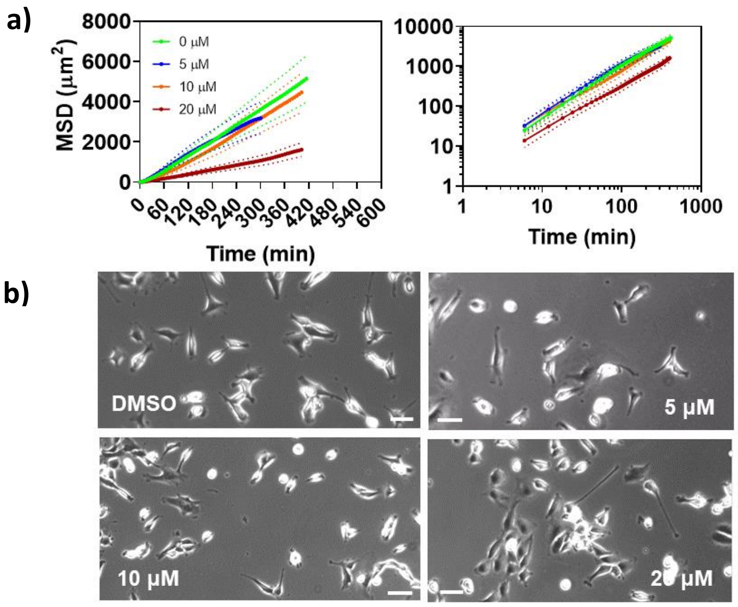

Figure S13: Analysis of NIH-3T3 fibroblasts migration in the presence of 1g. (a) plot of the MSD (linear-linear, on the left, and Log-Log, on the right) under different conditions. Only for a concentration of 20 µM we observe a decrease of the explored area by cells; (b) phase contrast images to show that 1g is not removing cell polarization for NIH-3T3 cells.

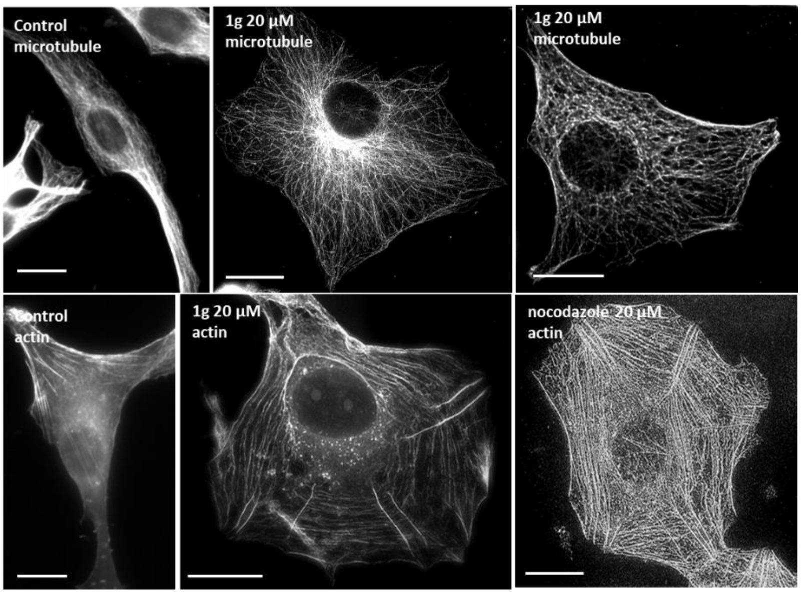

Figure S14: Immunofluorescence images of control U87MG cells and after incubation for 24 h in 20 µM 1g. Cells have been stained for actin and microtubules. Cells that are not blocked in the mitotic phase flatten on the surface developing a big structure of stress fibers but microtubules are still polimerized. The stress fiber organization is very similar, after 24h, to the structure assumed by U87MG cells exposed to a 20 µM concentration of nocodazole. (bar = 10 µM in all images)

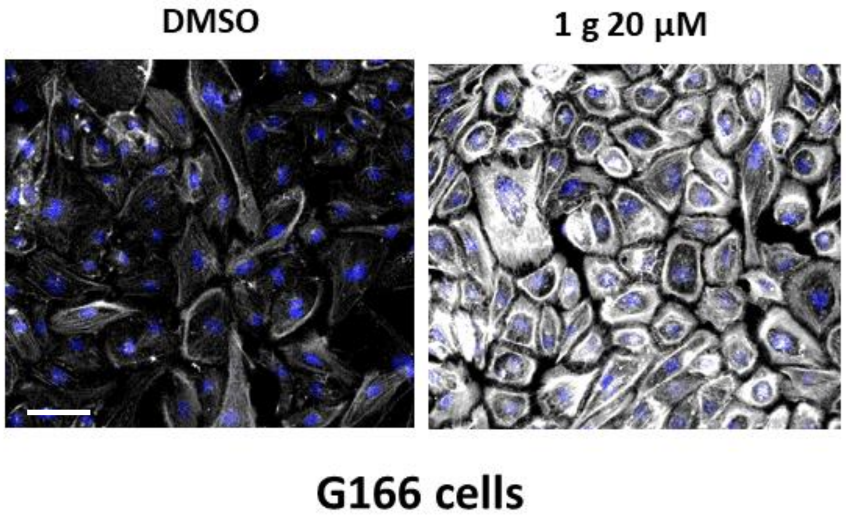

Figure S15: Immunofluorescence of G166 cells exposed to a 20 µM concentration of 1g. On the left the cells exposed to the vehicle are reported (blue → Dapi, white → F-actin). On the right the cells are exposed to 1g for 15 minutes (bar = 50 µm).

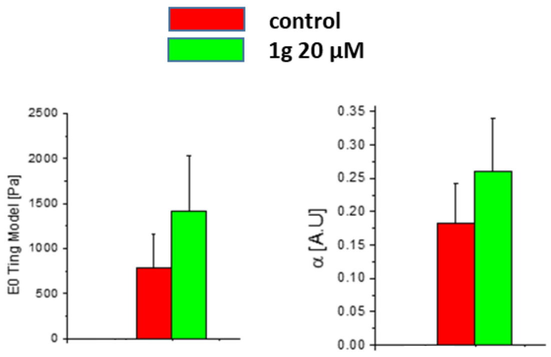

Figure S16: Analysis of the elastic and viscous contributions for U87MG control cells and the same type of cells exposed to 1g 20 µM for 24h. The analysis is based on the Ting model exploiting single approach/retract curves to obtain both contributions. E_0 represents the instantaneous Young modulus value whereas the value is a parameter accounting for the fluid or elastic properties of a body (α = 0, purely elastic material, α =1, purely viscous material).

Movies legend

Movie S1: Growth and expansion of a spheroid of U87MG cells embedded in a Matrigel®matrix. The movie, apart a time interval (see Figure 1), represents the growth over 68 hours and the spheroid is in DMSO control conditions. The boundaries of the growing spheroid have been highlighted using the Analyze_Spheroid_Cell_Invasion_In_3D_Matrix tool from FIJI.

Movie S2: Growth and expansion of a spheroid of U87MG cells embedded in a Matrigel®matrix with 20 µM 1g. The movie, apart a time interval (see Figure 1), represents the growth over 68 hours. The boundaries of the growing spheroid have been highlighted using the Analyze_Spheroid_Cell_Invasion_In_3D_Matrix tool from FIJI.

Movie S3: Higher magnification of a spheroid of U87MG cells embedded in a Matrigel®matrix in DMSO control conditions. The movie, apart a time interval (see Figure 1), represents the migration of U87MG cells over 68 hours.

Movie S4: Higher magnification of a spheroid of U87MG cells embedded in a Matrigel®matrix with 20 µM 1g. The movie, apart a time interval (see Figure 1), represents the migration of U87MG cells over 68 hours.

Movie S5: Time-lapse imaging of a spheroid of U87MG cells embedded in a Matrigel®matrix with 5µm diameter latex beads in DMSO control conditions. The sequence represents the displacement field of the beads due to contractility of the spheroid over a time interval of 46 hours.

Movie S6: Time-lapse imaging of a spheroid of U87MG cells embedded in a Matrigel®matrix with 5µm diameter latex beads and with 20 µM 1g. The sequence represents the displacement field of the beads due to contractility of the spheroid over a time interval of 46 hours.

Movie S7: Time-lapse imaging of a spheroid of U87MG cells in DMSO control conditions aspirated in a micropipette. After a pressure step of 1960 Pa, the pressure is released and the spheroid projection relaxes. The pressure step is kept constant for 40 minutes and then released. (bar = 50 µm)

Movie S8: Time-lapse imaging of a spheroid of U87MG cells in 20 µM 1g aspirated in a micropipette. After a pressure step of 1960 Pa, the pressure is released and the spheroid projection relaxes. The pressure step is kept constant for 40 minutes and then released. (bar = 50 µm)

Movie S9: Time-lapse (1 image every 4 minutes) imaging of a U87MG and NIH/3T3 co-culture. Before adding 1g to the culture medium both types of cells are elongated but, after the insertion of 20 µM 1g, only U87MG cells get round whereas 3T3 cells have a negligible change of their shape. The complete sequence corresponds to about 9 hours.

Movie S10- Time-lapse imaging (1 image every 4 minutes) of the seeding process of U87MG in the absence (left column) and in the presence (right column) of 20 µM 1g. The sequences correspond to an overall time of about 5 hours.

Movie S11- Time-lapse imaging (1 image every 4 minutes) of the wash-out process (the frame corresponding to the wash-out is evident from a sudden change of the alignment). U87MG cells are initially in DMSO and after a few frames 1g to a final concentration of 20 µM is injected.

Movie S12- Examples of attempts by U87MG cells in 20 µM 1g to exit mitosis.

Movie S13: Time-lapse sequences of fluorescence images of U87MG cells exposed to 20 µM 1g. Tubulin has been stained using Sir-tub as a live fluorescent marker. The sequences show examples of mitosis with an

altered mitotic fuse. One image (look at the initial frame) is the overlap of fluorescence and phase contrast signals.

Movie S14: Time-lapse sequences (1 frame every 4 miutes) of U87MG cells exposed to 20 µM blebbistatin (on the left) and 20 µM blebbistatin+20 µM 1g (on the right). (bar = 100 µm)

Movie S15: Time-lapse sequences (1 frame every 4 miutes) of U87MG cells exposed to 20 µM Y-27632 (on the left) and 20 µM Y-27632 +20 µM 1g (on the right). (bar = 100 µm)

Movie S16: Polarity loss and continuous attempts to polarize corresponding to the formation and retraction of pseudopods of U87MG cells exposed to 20 µM 1g.
